# Psychometric Properties of the Emotional Exhaustion Scale (ECE) in Chilean Higher Education Students

**DOI:** 10.3390/ejihpe12010005

**Published:** 2022-01-10

**Authors:** Jonathan Martínez-Líbano, María-Mercedes Yeomans, Juan-Carlos Oyanedel

**Affiliations:** 1 Facultad de Educación y Ciencias Sociales, Universidad Andrés Bello, Santiago 8370035, Chile; juan.oyanedel@unab.cl; 2 Doctorado en Educación, Universidad Internacional Iberoamericana, Campeche 00613, Mexico; 3 Facultad de Educación, Universidad de Las Américas, Viña del Mar 2520000, Chile

**Keywords:** emotional exhaustion, mental health, university students

## Abstract

The main objective of this study was to estimate the psychometric properties of the Emotional Fatigue Scale (ECE) in a sample of 1308 Chilean university students and confirm the unifactorial structure of the scale. Exploratory and confirmatory factor analyses were carried out. The ECE assessment had an internal consistency of 0.893 (Cronbach’s Alpha). An exploratory factor analysis with Varimax rotation and a confirmatory analysis were performed, obtaining the factor that explains 52.3% of the variance. The results indicated that the ECE has adequate psychometric properties for use with higher education students in Chile. The ECE scale has good psychometric properties to be applied in the Chilean university context. Its usage may be very relevant to contribute to higher education institutions to emphasize students’ mental health and prevent possible severe pathologies in future professionals. It is suggested to use the ECE scale together with the EES-Int, which is the only interpretation table for this instrument.

## 1. Introduction

Emotional exhaustion is a major problem among students in higher education and a public health problem in general [1,2,3,4,5,6]. Higher education is a challenging context where students, particularly those with low resources, are susceptible to emotional exhaustion [3,7]. The impact of poverty on students’ academic achievement is significant; students may face challenges with their cognitive and literary ability and often begin school academically and socioeconomically behind their peers from higher-income backgrounds [8]. Emotional exhaustion or burnout refers to the situation in which people feel that they can no longer give more of themselves on an affective level; it is a situation of depletion of one’s energy or emotional resources [9]. In recent years, emotional exhaustion among adolescent and university students has acquired broader importance in national educational policy in many countries [10,11,12,13,14,15]. Moreover, emotional exhaustion develops from an acute to a chronic problem when faced with persistently stressful circumstances [2,3,4,16]. One example is the effects of a pandemic such as COVID-19 on the mental health of Chilean students [1,3,4]; 15% of Chilean university students had severe mental health problems [17], moderate depression has increased from 17.7% to 20.7%, severe depression has increased by 5.2%, severe anxiety has risen from 16.7% to 26.4%, and severe stress has increased from 9.4% to 15.9% [18].

A student’s level of burnout directly impacts that student’s level of efficiency and productivity [11]. More than one-third of college students experience detrimental imbalances between the effort they put into their studies and the rewards they receive in return [19]. The consequences of emotional exhaustion in students are as complex as other factors of mental distress, such as those related to smoking and alcohol consumption or an unhealthy lifestyle [6]. Emotional exhaustion can also lead to depression [20] and anxiety [21], feelings of abandonment and Internet addiction, and compromised satisfaction with life in general, and success in educational pathways [22]. We can add practical consequences such as dropping out of school [19], potentially leading to sleep disorders, depression, and even suicidal thoughts and ideation in this age group [2,23]. Together with emotional exhaustion, drugs and alcohol consumption to reduce stress cause concern [24]. A student’s performance can be seriously affected by the various emotional, behavioral, cognitive, and physiological reactions caused by burnout [11].

Several tests measure burnout or emotional exhaustion as a primary or secondary endpoint. The Maslach Burnout Inventory (MBI) instrument evaluates three areas: personal fulfillment at work (eight items), emotional exhaustion (nine items), and depersonalization (five items) [25,26,27]. On the other hand, the emotional exhaustion scale of the Copenhagen Psychosocial Questionnaire (CPQ) was developed by a consortium of health and wellbeing researchers in the workplace [28]. The scale consists of four Likert-scaled items where participants rate how they currently feel on a 5-point scale, whose estimated mean reliability was 0.77 [29]. Finally, the Emotional Fatigue Scale (ECE) is a specific scale that considers the last 12 months of student life; the items are inspired by the MBI emotional exhaustion scale and include Freudenberger’s concept of burnout. To this base were added items specially designed to evaluate university students’ fatigue or emotional exhaustion, which derived from the level of demand and effort to overcome their studies [30].

Although the MBI was validated for use in Chilean adults in 2005, its primary purpose is not to assess emotional exhaustion in university students [31]. On the other hand, the CPC focuses only on the work environment [28]. Emotional exhaustion needs to be investigated in depth since it is the most relevant burnout variable. It is, for some authors, the most obvious, complex, and the most widely reported and analyzed manifestation, which is associated with many psychological and psychiatric pathologies [32,33,34,35,36]. Concerning the university population, emotional exhaustion is one of the main concerns of the educational system [16]. Having a scale with good psychometric properties to be measured in the Chilean university context is very relevant [30] to contribute to higher education institutions’ authorities to emphasize students’ mental health and prevent possible severe pathologies in future professionals [1]. Therefore, this study aims to estimate the psychometric properties of the ECE in Chilean university students.

## 2. Materials and Methods

### 2.1. Participants

The sample was nonprobabilistic, composed of 1308 students from Chilean institutions of higher education, who met the following inclusion criteria, accepted the informed consent, and were studying in an institution of higher education. Inclusion criteria: University students and former students who graduated less than one year before the evaluation, Chilean, and enrolled in Chilean higher education institutions that had signed the informed consent. Exclusion criteria: All incomplete questionnaires were excluded. Concerning ages, the mean and standard deviation were 26.86 ± 8.10. The coefficient of variation (CV) was 35.30. The sample was nonprobabilistic, composed of 1308 students from Chilean institutions of higher education, aged between 17 and 69 years (≤17: 0.2%; 18–20: 26.1%; 21–29: 49.3%; 30–39: 17.2%; 40–49: 5%; 50–59: 2.2%; 60–69: 0.2%). Concerning gender, 20.9% were male, and 79.1% were female. About majors, the sample consisted of health sciences (33.4%), social sciences (15.4%), natural sciences (12.4%), education (11.7%), business and administration (9.3%), engineering and technology (6%), communication (5%), architecture and arts (1%), and legal sciences (5.8%). Regarding the academic progress of the students, 27.2% of them were in the first year, 23.3% in the second year, 21.6% in the third year, 16.7% in the fourth year, 8.1% in the fifth year, and 3.1% in the process of graduation.

### 2.2. Instruments

For this study, a questionnaire was elaborated, which was validated through expert judgment. The first section included demographic questions such as age, gender, and academic program. The second section included general questions regarding the perception of students’ own mental health, such as: “Do I think my mental health has worsened?”; “At this time, I have presented symptoms of stress?”; and “At this time, I have presented depressive symptoms?”. The third section consisted of the emotional exhaustion scale (ECE) (Appendix A) [30]. It is a specific scale that considers the last 12 months of students’ lives. It was inspired by the MBI emotional exhaustion scale [37] and Freudenberger’s concept of burnout. The ECE is a unidimensional scale of 10 items. The items are scored from 1 to 5, depending on the occurrence of what the statement says: 1 = rarely, 2 = few times, 3 = sometimes, 4 = frequently, and 5 = always. The score obtained on the ECE ranges from 10 to 50 points. Its level of internal consistency (an alpha coefficient of 0.893) and satisfactory item homogeneity (mean interitem correlation = 0.33). The ECE results are interpreted by the Emotional Exhaustion Scale Interpretation Table (EES-Int) in the cognitive, emotional, and social-interactive dimensions [3]. The questionnaire applied electronically includes, as a first step, the signing of informed consent for data collection. The recollected data were demographic and anonymous. The study was conducted under the authorization of the Bioethics Committee of Universidad Andrés Bello, under resolution 90660/2020. The whole study was performed under the latest version of the Helsinki declaration.

### 2.3. Data Analysis

The data were analyzed with SPSS version 25 for Windows [38], *p* value ≤ 0.001. The instrument’s reliability was estimated by calculating Cronbach’s alpha internal consistency [39]. We calculated the corrected item correlation and the alpha coefficient for the ten items. An exploratory factor analysis was performed with the extraction of principal components and Varimax rotation. For the confirmatory factor analysis, SPSS AMOS was used [40]. We included the Kaiser–Meyer–Olkin sample adequacy measure (KMO) and Barlett’s test of sphericity, measuring the approximate Chi-square (362.386), degree of freedom (df) (35), and significance (*p* ≤ 0.001) [41,42]. Regarding the factors, the discrepancy between the model and the actual data of the sample was measured through an Explained Variance analysis. Finally, we analyzed the absolute fit measures (Chi-square, RMSEA), incremental fit measures (CFI, TLI, IFI, NFI), and parsimony fit measures (PRATIO, PCFI, PNFI, AIC).

## 3. Results

### 3.1. Instrument Reliability

Cronbach’s Alpha reliability analysis was performed for the entire scale to calculate the instrument’s reliability. Table 1 shows the correlations between the items and the total score at 0.03. The total reliability of the instrument is 0.893, including all the items. In addition, reliability analysis was performed for each of the ten items (see Table 1).

### 3.2. Factor Analysis of the Scale

An exploratory factor analysis was performed to estimate the validity of the ECE. As shown in Table 2, the KMO sample adequacy measure resulted in a value of 0.933. Likewise, Bartlett’s test of sphericity is significant (x^2^ = 5765.512097; *p* ≤ 0.001). These results indicate that the necessary conditions are met to proceed with the factor analysis.

**Figure 1:**
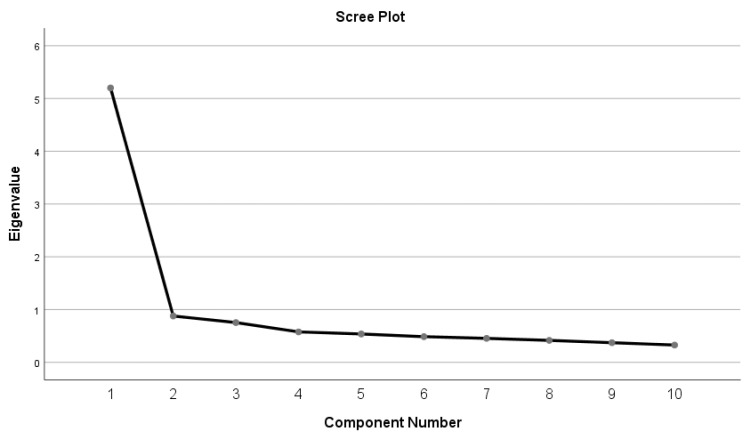
ECE Scree Plot. Considering that the results show a structure similar to previously reported validations [30,43] and Cronbach’s alpha results suggest that eliminating items would reduce its total value, the original scale structure was maintained.

Table 3 shows the factors yielded by the principal component analysis, the percentage of the individual variance of each factor, and the percentage of accumulated variance. Consistent with Ramos et al., a single factor with an eigenvalue more significant than one is extracted [30]. Looking at Figure 1, which corresponds to the sedimentation plot, the previous result tends to be confirmed.

### 3.3. Factorial Confirmatory Analysis

**Figure 2:**
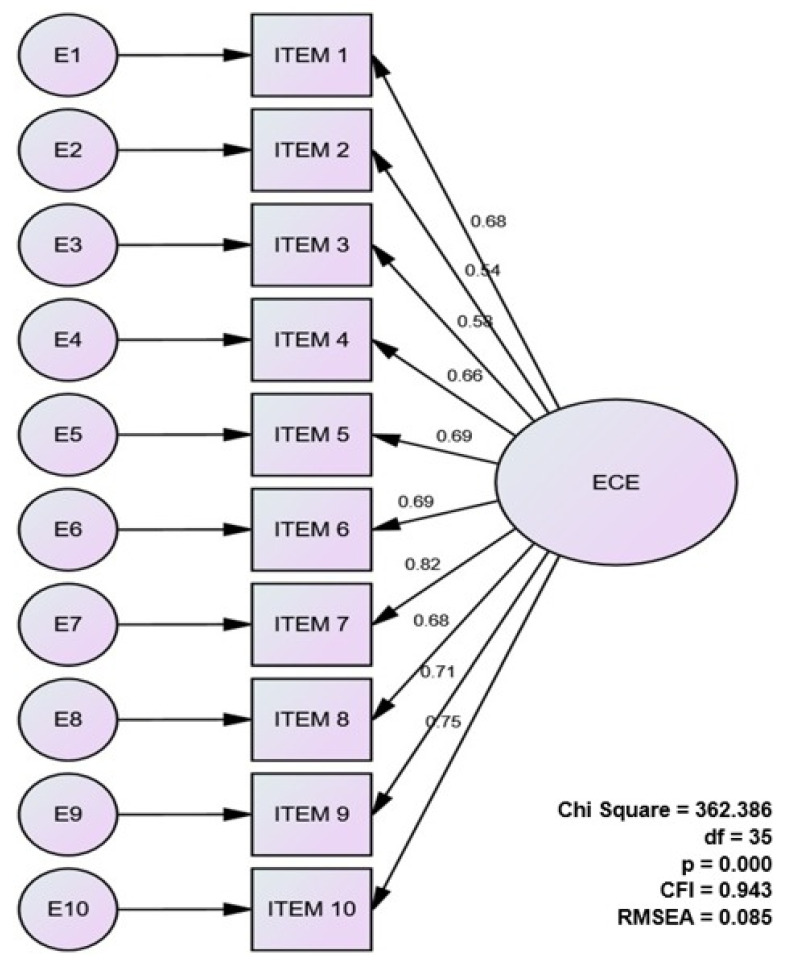
Confirmatory factor analysis graphic.

In the analysis presented in Figure 2, all parameters were significant, and the indicators of the model presented fair values. Table 4 shows the goodness-of-fit results of the hypothetical model, which were sufficient to support the unifactorial structure of the scale. Positive results were identified in the CFI (0.943) and TLI (0.927), RMSEA values (0.85) and chi-square test (χ^2^ = 362.386, *p* < 0.000, χ^2^/gl = 10.354). Thus, the criteria required to accept an adequate value are met. All the indicators of the model (Table 4) showed fair values. The fit indexes showed acceptable values. The CFI is close to 0.95 showing a reasonable fit [44], whereas the RMSEA (0.85) presents an acceptable fit considering the sample size and model specification [45].

In Table 5, it is observed that the model meets the scalar invariance criterion. The means of the latent factors can be compared since there is a Δ CFI < 0.010 and a Δ RMSEA < 0.015 [45].

## 4. Discussion

The main objective of this study was to estimate the psychometric properties of the Emotional Fatigue Scale (ECE) in a sample of 1308 Chilean university students and confirm the unifactorial structure of the scale. The original psychometric properties of the scale concerning internal consistency were a Cronbach’s alpha of 0.83 and a unifactorial structure that explains 40% of the total variance. The instrument originated in Spain and was validated with students aged between 18 and 33 years [30]. When comparing our results with those reported by Ramos et al. (2005), we can observe that the variance explained in the exploratory factor analysis is higher than that reported by the authors of the ECE. In this study, the variance explained in the exploratory factor analysis was 52% and 94% in the confirmatory factor analysis. Our study’s internal consistency (α = 0.893) is slightly higher than the original (α = 0.83). Although the goodness-of-fit indicators (Table 4) indicate that the model is susceptible to improvement, the percentage of variance explained suggests that working with a total ECE score is adequate. Thus, the unifactorial structure of the scale is confirmed. It can be said that the scale structure is maintained to allow comparative analysis from the above. Still, it is necessary to perform further studies to analyze factorial invariance.

This scale has been applied in different higher education students [3,46,47,48,49]. Moreover, there are many studies in mental health on the studied population [2,3,4,5]. Even though Chilean policies have been successfully adapting to emerging needs [5,50], the scale has not been used until now in Chile, and we believe its validation will contribute towards that.

In our study, the Chilean validation maintains the psychometric structure for the instrument. The issue of the variance explained by the exploratory factor analysis (EFA) is diluted for two reasons: first, because only one factor has a high eigenvalue, meaning it does not make sense to increase the number of factors; despite integrating sensitivity to the instrument, it reduces its specificity [51,52]. On the other hand, when reviewing the Confirmatory Factor Analysis (CFA) data, it is possible to see a proper fit in its original version. Keeping the original version, which has acceptable psychometric properties, would allow the comparison between students from different countries [53]. This should be tested by employing invariance analysis in future studies [54].

The appropriate standardization of a given assessment instrument makes it possible to develop national norms for the valid interpretation of the meaning of a given person’s scores on the standardized test [55]. Therefore, it is very relevant to study the adaptation of the scale for Chilean undergraduate students.

Concerning the Emotional Fatigue Scale (ECE), internal consistency in our sample of 1308 participants was 0.893. Whether the reliability of a given assessment instrument is expressed in terms of an alpha coefficient, test–retest, interexaminer, or temporal reliability coefficient, it is helpful to develop guidelines for distinguishing clinically meaningful levels from those that may not be. Given the caveats regarding item ceiling and item floor effects and the need to consider alpha coefficients in the broader context of other types of reliability assessments (e.g., interexaminer), Cicchetti suggested guidelines. When the size of the alpha coefficient or other measure of internal consistency is below 0.70, the level of clinical significance is unacceptable; when it is between 0.70 and 0.79, the level of clinical significance is fair; when it is between 0.80 and 0.89, the level of clinical significance is good; and when it is 0.90 and above, the level of clinical significance is excellent [55,56]. Based on the above, we can say that the Emotional Fatigue Scale (ECE) has a good level of clinical significance. The Cronbach’s alpha of 0.893 found in our study is higher than that of other studies taken as a reference on the same subject (0.853 [57] 0.874 [58], 0.83 [59]).

This research confirms the principal component factor analysis, yielding a single factor with the ten items that make up the ECE. In this sense, the ECE scale has good psychometric behavior, presenting excellent evidence of factorial validity and good reliability indicators. The ECE is short, with only ten items, making easy to administer and score. In that case, it seems that the ECE scale is suitable for measuring emotional exhaustion in higher education students.

When comparing our study to the analysis of psychometric properties in Mexico [43], the latter was carried out with 506 psychology students from two universities. Exploratory and confirmatory factor analyses were performed, where an internal reliability of 0.90 was determined, confirming the unifactorial structure of the ECE and providing evidence of the validity of the scale.

Regarding the analysis of the psychometric properties of the ECE carried out in Peru [46], a similar sample of 448 psychology students from a private university was evaluated for reliability. Cronbach’s alpha coefficient of 0.87 was adequate, proving the unifactoriality of the scale.

Although our study presents a more extensive and more diverse sample (1308 participants from different majors), we can still verify good internal reliability and a correct fit with a Cronbach’s alpha coefficient of 0.893, proving the unifactoriality of the scale.

Emotional exhaustion is a construct that should continue to be studied in the university context. In students, it has been correlated with anxiety, depression [60], stress [61,62], and some personality traits [30], which could affect the normative development of university students’ lives.

In summary, the ECE has characteristics that make it very valuable for assessing emotional exhaustion in university students. It is a self-report instrument with excellent psychometric properties, aimed at a specific population such as higher education students. This instrument can help students, teachers, academics, and university authorities to detect and prevent possible psychological disorders in university students in their mental health.

Likewise, it is essential to develop an instrument to measure emotional exhaustion in teachers since it has been associated with their self-efficacy (as an enduring self-regulatory trait) [63], mood [16], stress [64], anxiety [65], and depression [60].

Keeping the original version of the ECE, which has acceptable psychometric properties, would allow the comparison between students from different countries, which should be tested by employing invariance analysis in future studies.

Although the study sample is large, it is not probabilistic. In future studies, the sample should be increased.

Concerning gender, the sample was not homogeneous between males and females (20.9% and 79.1%, respectively); therefore, the studio may not represent the underrepresented gender.

Finally, the sample used was composed of university students, representing a very limited and special population considering their high level of education compared to the country population.

## 5. Conclusions

This study describes the psychometric properties of the emotional exhaustion scale (ECE) in a sample of Chilean university students. The properties of this version are good, and the results support the reliability and validity related to the construct of emotional exhaustion measured by the ECE. The ECE evaluation had an internal consistency of 0.893. An exploratory factor analysis with Varimax rotation and a confirmatory analysis obtained a unifactorial model that explains 52.3% of the variance. The confirmatory factor analysis was 94%. Therefore, the study of the results suggests that the emotional exhaustion scale has a good adequate psychometric property for higher education students in Chile. Consequently, the ECE is a reliable instrument to measure emotional exhaustion in Chilean university students. To better understand the mental health of Chilean university students, this instrument should be applied to other samples and correlated with other phenomena such as stress, anxiety, and depression.

The ECE scale has good psychometric properties to be applied in the Chilean university context. Its usage may be very relevant to contribute to higher education institutions to emphasize students’ mental health and prevent possible severe pathologies in future professionals.

It is suggested to use the ECE scale together with the EES-Int [3], which is the only interpretation table for this instrument.

## Figures and Tables

**Table 1:** Corrected item correlation and Cronbach’s alpha if the item is removed.

Component	Corrected Item Correlation	Cronbach’s Alpha If the Item Is Removed
I	0.641	0.882
II	0.518	0.892
III	0.553	0.888
IV	0.62	0.883
V	0.655	0.881
VI	0.656	0.881
VII	0.762	0.873
VIII	0.634	0.883
IX	0.659	0.881
X	0.699	0.878

**Table 2:** Factor analysis application conditions.

KMO		0.933
Sphericity Bartlett’s test	Approximate Chi-square	5765.512097
	df	45
	*p*-value	0.000

Df: degree of freedom; KMO: Kaiser–Meyer–Olkin measure; *p*-value: significance.

**Table 3:** Eigenvalues and percentage of variance explained by each factor.

Initial Eigenvalues			
Component	Total	Variance%	Accumulate%
**1**	5.200	52.003	52.003
**2**	0.878	8.777	60.780
**3**	0.754	7.537	68.317
**4**	0.577	5.765	74.082
**5**	0.537	5.370	79.452
**6**	0.486	4.863	84.315
**7**	0.453	4.533	88.848
**8**	0.416	4.159	93.008
**9**	0.372	3.718	96.726
**10**	0.327	3.274	100.000

**Table 4:** Confirmatory factor analysis goodness-of-fit indicators.

Absolute Fit Measures	Chi-Square	0
	RMSEA	0.85
Incremental Fit Measures	CFI	0.943
	TLI	0.927
	IFI	0.943
	NFI	0.937
Parsimony Fit Measures	PRATIO	0.778
	PCFI	0.733
	PNFI	0.729
	AIC	422.386

**Table 5:** Invariance models.

Model	χ^2^ (gl)	CFI	RMSEA [LCI-HCI]	SRMR	Δ CFI	Δ RMSEA
Configural	330.664 *** (68)	0.953	0.054 [0.049–0.060]	0.041		
Metric	339.968 *** (77)	0.953	0.051 [0.046–0.057]	0.043	0.000	−0.003
Scalar	366.677 *** (86)	0.949	0.050 [0.045–0.055]	0.043	−0.004	−0.001

Note. *** *p* < 0.001.

## Data Availability

Data sets analyzed or generated during the study are available for sharing if needed.

## Appendix A

EMOTIONAL EXHAUSTION SCALE (RAMOS, 2005)

Below you will find a series of questions that refer to your way of thinking, feeling, and acting concerning your studies. Read each sentence and answer according to the last 12 months of your student’s life by marking with an X according to the following rating scale:

**Item**	**Rarely**	**Few Times**	**Sometimes**	**Often**	**Always**
Tests or evaluations cause me excessive stress.					
I think I try too hard for the little I get out of it.					
I feel down in the dumps, kind of sad, for no apparent reason.					
There are days when I don’t sleep well because of studying.					
I have headaches and other discomforts that affect my performance.					
There are days when I feel more exhausted, and I lack the energy to concentrate.					
I feel emotionally drained by my studies.					
I feel tired at the end of the day.					
Working and/or studying with evaluations in mind causes me stress.					
I lack time, and I feel overwhelmed by my studies.					
